# (Dimethyl sulfoxide-κ*O*){4,4′,6,6′-tetra-*tert*-butyl-2,2′-[1,2-dicyano­ethene-1,2-diylbis(nitrilo­methyl­idyne)]diphenolato-κ^4^ *O*,*N*,*N*′,*O*′}zinc(II) acetonitrile monosolvate

**DOI:** 10.1107/S160053681100359X

**Published:** 2011-02-09

**Authors:** E. S. Aazam, Seik Weng Ng, Edward R. T. Tiekink

**Affiliations:** a Department of Chemistry, Girls Campus, King Abdulaziz University, PO Box 6171, Jeddah 21442, Saudi Arabia; b Department of Chemistry, University of Malaya, 50603 Kuala Lumpur, Malaysia

## Abstract

The Zn atom in the title acetonitrile solvate, [Zn(C_34_H_42_N_4_O_2_)(C_2_H_6_OS)]·CH_3_CN, exists in a distorted square-pyramidal geometry with the basal plane defined by the N_2_O_2_ atoms of the tetra­dentate Schiff base and with the dimethyl sulfoxide O atom in the apical position. The tetra­dentate mode of coordination of the Schiff base ligand leads to a five-membered ZnN_2_C_2_ chelate ring which adopts an envelope conformation with the Zn atom at the flap, and two six-membered ZnOC_4_N chelate rings, one of which is approximately planar (r.m.s. deviation = 0.054 Å) but the other has significant puckering (r.m.s. deviation = 0.203 Å).

## Related literature

For background to metal salicylaldiminato complexes as optoelectronic materials, see: Liuzzo *et al.* (2010 ▶); Shirai *et al.*, (2000 ▶). For background to zinc complexes as organic light-emitting diodes, see: Chen *et al.* (2009 ▶). For related structures, see: MacLachlan *et al.* (1996 ▶). For geometrical analysis, see: Addison *et al.* (1984 ▶).
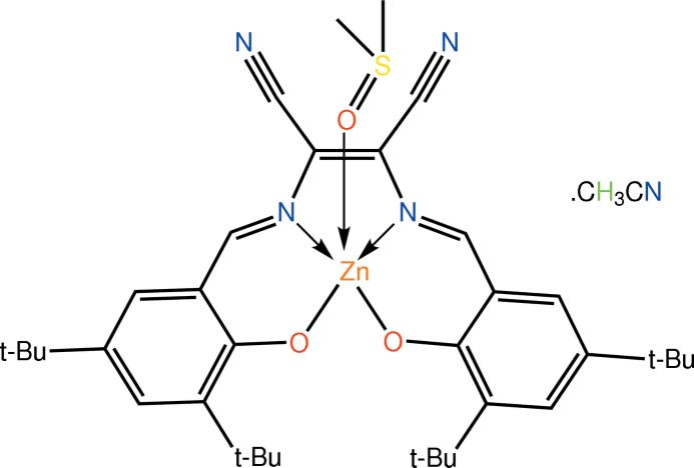


## Experimental

### 

#### Crystal data

[Zn(C_34_H_42_N_4_O_2_)(C_2_H_6_OS)]·C_2_H_3_N
*M* *_r_* = 723.27
Monoclinic, 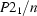

*a* = 12.3288 (7) Å
*b* = 17.7043 (9) Å
*c* = 17.3932 (9) Å
β = 92.4391 (8)°
*V* = 3793.0 (3) Å^3^
*Z* = 4
Mo *K*α radiation
μ = 0.74 mm^−1^
*T* = 100 K
0.45 × 0.30 × 0.10 mm

#### Data collection

Bruker SMART APEX CCD diffractometer
Absorption correction: multi-scan (*SADABS*; Sheldrick, 1996 ▶) *T*_min_ = 0.603, *T*_max_ = 0.746
35700 measured reflections
8699 independent reflections
7157 reflections with *I* > 2σ(*I*)
*R*_int_ = 0.041

#### Refinement

*R*[*F* ^2^ > 2σ(*F* ^2^)] = 0.032
*wR*(*F* ^2^) = 0.080
*S* = 1.02
8699 reflections
448 parameters
H-atom parameters constrained
Δρ_max_ = 0.56 e Å^−3^
Δρ_min_ = −0.29 e Å^−3^

### 

Data collection: *APEX2* (Bruker, 2008 ▶); cell refinement: *SAINT* (Bruker, 2008 ▶); data reduction: *SAINT*; program(s) used to solve structure: *SHELXS97* (Sheldrick, 2008 ▶); program(s) used to refine structure: *SHELXL97* (Sheldrick, 2008 ▶); molecular graphics: *ORTEP-3* (Farrugia, 1997 ▶), *DIAMOND* (Brandenburg, 2006 ▶); software used to prepare material for publication: *publCIF* (Westrip, 2010 ▶) and *PLATON* (Spek, 2009 ▶).

## Supplementary Material

Crystal structure: contains datablocks global, I. DOI: 10.1107/S160053681100359X/hb5794sup1.cif

Structure factors: contains datablocks I. DOI: 10.1107/S160053681100359X/hb5794Isup2.hkl

Additional supplementary materials:  crystallographic information; 3D view; checkCIF report

## Figures and Tables

**Table 1:** Selected bond lengths (Å)

Zn—O1	1.9470 (11)
Zn—O2	1.9390 (11)
Zn—O3	2.0467 (12)
Zn—N1	2.0939 (14)
Zn—N2	2.1001 (13)

**Fig. 1.**
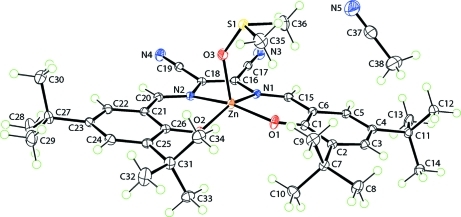
The molecular structure of (I) showing displacement ellipsoids at the 50% probability level.

## supplementary crystallographic information

### Comment

Metal complexes with salicylaldiminato ligands are promising materials for optoelectronic applications due to their outstanding photo- and electro-luminescent properties (Liuzzo *et al.*, 2010; Shirai *et al.*, 2000). One of the main appeals of this class of coordination complexes is that molecular engineering permits systematically the optimizing of spectroscopic and chemical properties. This chemical flexibility allows for the design of systems that respond to specific environmental variables. Recently, zinc complexes have been introduced to OLED's (organic light-emitting diodes) and recognized as useful electron transport materials (Chen *et al.*, 2009). The above motivated the synthesis and structural characterization of the title complex, (I).

The Zn atom in (I), Fig. 1, is tetracoordinated by the N_2_O_2_ donor atoms of the tetradentate Schiff-base ligand and the O atom derived from the dimethyl sulfoxide ligand, Table 1; the asymmetric unit is completed by a non-coordinating acetonitrile molecule. The resulting N_2_O_3_ donor set is based on a square pyramidal arrangement with the dimethyl sulfoxide-O3 atom occupying an axial site. The value of τ = 0.16 compares with τ = 0 and 1.0 for ideal square pyramidal and trigonal bipyramidal geometries, respectively (Addison *et al.*, 1984). The r.m.s. deviation of the O1, O2, N1 and N2 atoms from their least-squares plane is 0.0836 Å and the Zn atom lies 0.3976 (7) Å out of the plane towards the O3 atom. The tetradentate mode of coordination of the Schiff-base leads to the formation of a five- and two six-membered rings. The former has a conformation based on an envelope on Zn (Spek, 2009). While the chelate ring involving the O1 atom is approximately planar (r.m.s. = 0.054 Å), there is significantly more distortion in the O2-containing chelate ring (r.m.s. = 0.203 Å). Schiff-base ligands derived from diaminomaleonitrile have been documented and shown to adopt comparable coordination modes towards transition metals (MacLachlan *et al.*, 1996).

### Experimental

A mixture of diaminemaleonitrile (0.1 g, 0.93 mmol), 3,5-di-*tert*-butyl-2-hydroxybenzaldehyde (0.1 g, 1.86 mmol), zinc(II) acetate dihydrate (0.2 g, 0.93 mmol) and ethanol (5 ml) were placed in a glass Petri dish and capped with a glass cover. The dish was placed in a microwave oven (700 W) and irradiated for 1 min. The reaction mixture was cooled and washed with 15 ml of ethanol. The purple solid was filtered off and washed with ethanol. Re-crystallization was by slow evaporation of an acetonitrile/dimethyl sulfoxide (90/10 *v*/*v*) solution which yielded purple blocks of (I). Yield: 70%. *M*.pt. > 623 K (dec.). ^1^H NMR (DMSO-d6, 500 MHz): δ = 1.25 (s, 18H, C(CH_3_)_3_), 1.47 (s, 18H, C(CH_3_)_3_), 2.06 (MeCN), 2.50 (DMSO), 7.24 (s, 2H, Ar—H), 7.43 (s, 2H, Ar—H), 8.58 (s, 2H, N═CH) p.p.m.. ^13^C NMR (DMSO-d6, 500 MHz): δ = 28.19, 29.80 (C(CH_3_)_3_), 32.61, 34.13 (C(CH_3_)_3_), 110.48, 116.85, 120.31, 128.10, 130.17, 134.20, 141.11, 161.68 and 172.33 p.p.m. IR: 2952, 2212 (C≡N), 1616 (C═N), 1569, 1519, 1433, 1372, 1170, 1154, 1119, 1032, 795, 656 cm^-1^. λ_max_ (DMSO, 10 ^-5^ mol *L*^-1^): 574, 501, 380, 375, 318, 245 nm.

### Refinement

Carbon-bound H-atoms were placed in calculated positions (C–H = 0.95 to 0.98 Å) and were included in the refinement in the riding model approximation, with *U*_iso_(H) set to 1.2–1.5*U*_equiv_(C). In the final refinement three low angle reflections evidently effected by the beam stop were omitted, *i.e*. (011), (101) and (110).

### Crystal data

**Table:**

[Zn(C_34_H_42_N_4_O_2_)(C_2_H_6_OS)]·C_2_H_3_N	*F*(000) = 1536
*M**_r_* = 723.27	*D*_x_ = 1.267 Mg m^−^^3^
Monoclinic, *P*2_1_/*n*	Mo *K*α radiation, λ = 0.71073 Å
Hall symbol: -P 2yn	Cell parameters from 9906 reflections
*a* = 12.3288 (7) Å	θ = 2.3–28.3°
*b* = 17.7043 (9) Å	µ = 0.74 mm^−^^1^
*c* = 17.3932 (9) Å	*T* = 100 K
β = 92.4391 (8)°	Block, purple
*V* = 3793.0 (3) Å^3^	0.45 × 0.30 × 0.10 mm
*Z* = 4	

### Data collection

**Table:**

Bruker SMART APEX CCD diffractometer	8699 independent reflections
Radiation source: fine-focus sealed tube	7157 reflections with *I* > 2σ(*I*)
graphite	*R*_int_ = 0.041
ω scans	θ_max_ = 27.5°, θ_min_ = 2.3°
Absorption correction: multi-scan (*SADABS*; Sheldrick, 1996)	*h* = −16→16
*T*_min_ = 0.603, *T*_max_ = 0.746	*k* = −23→23
35700 measured reflections	*l* = −22→22

### Refinement

**Table:**

Refinement on *F*^2^	Primary atom site location: structure-invariant direct methods
Least-squares matrix: full	Secondary atom site location: difference Fourier map
*R*[*F*^2^ > 2σ(*F*^2^)] = 0.032	Hydrogen site location: inferred from neighbouring sites
*wR*(*F*^2^) = 0.080	H-atom parameters constrained
*S* = 1.02	*w* = 1/[σ^2^(*F*_o_^2^) + (0.036*P*)^2^ + 1.6768*P*] where *P* = (*F*_o_^2^ + 2*F*_c_^2^)/3
8699 reflections	(Δ/σ)_max_ = 0.002
448 parameters	Δρ_max_ = 0.56 e Å^−^^3^
0 restraints	Δρ_min_ = −0.29 e Å^−^^3^

### Special details

**Table:**

Geometry. All s.u.'s (except the s.u. in the dihedral angle between two l.s. planes) are estimated using the full covariance matrix. The cell s.u.'s are taken into account individually in the estimation of s.u.'s in distances, angles and torsion angles; correlations between s.u.'s in cell parameters are only used when they are defined by crystal symmetry. An approximate (isotropic) treatment of cell s.u.'s is used for estimating s.u.'s involving l.s. planes.
Refinement. Refinement of *F*^2^ against ALL reflections. The weighted *R*-factor *wR* and goodness of fit *S* are based on *F*^2^, conventional *R*-factors *R* are based on *F*, with *F* set to zero for negative *F*^2^. The threshold expression of *F*^2^ > 2σ(*F*^2^) is used only for calculating *R*-factors(gt) *etc*. and is not relevant to the choice of reflections for refinement. *R*-factors based on *F*^2^ are statistically about twice as large as those based on *F*, and *R*- factors based on ALL data will be even larger.

### Fractional atomic coordinates and isotropic or equivalent isotropic displacement parameters (Å^2^)

**Table:**

	*x*	*y*	*z*	*U*_iso_*/*U*_eq_	
Zn	0.516854 (14)	0.654898 (11)	0.558637 (10)	0.01242 (6)	
S1	0.41362 (3)	0.82176 (2)	0.50384 (2)	0.01635 (9)	
O1	0.63893 (9)	0.70123 (7)	0.61554 (7)	0.0184 (3)	
O2	0.44436 (9)	0.61428 (7)	0.64636 (6)	0.0167 (2)	
O3	0.41323 (9)	0.73722 (7)	0.51757 (7)	0.0198 (3)	
N1	0.61797 (11)	0.64054 (7)	0.46602 (8)	0.0129 (3)	
N2	0.42744 (10)	0.57668 (7)	0.49091 (7)	0.0121 (3)	
N3	0.64563 (12)	0.60956 (9)	0.27021 (9)	0.0224 (3)	
N4	0.34261 (12)	0.51244 (9)	0.30973 (9)	0.0221 (3)	
N5	0.63439 (16)	1.00678 (11)	0.54854 (14)	0.0500 (6)	
C1	0.73798 (13)	0.71491 (9)	0.59846 (9)	0.0133 (3)	
C2	0.81352 (12)	0.74304 (9)	0.65753 (9)	0.0127 (3)	
C3	0.91590 (13)	0.76392 (9)	0.63645 (9)	0.0136 (3)	
H3	0.9635	0.7851	0.6751	0.016*	
C4	0.95562 (12)	0.75609 (9)	0.56135 (9)	0.0128 (3)	
C5	0.88612 (12)	0.72505 (9)	0.50676 (9)	0.0129 (3)	
H5	0.9107	0.7177	0.4563	0.015*	
C6	0.77787 (12)	0.70320 (9)	0.52280 (9)	0.0130 (3)	
C7	0.78027 (12)	0.74618 (9)	0.74153 (9)	0.0138 (3)	
C8	0.87537 (14)	0.77112 (11)	0.79528 (10)	0.0212 (4)	
H8A	0.9355	0.7353	0.7914	0.032*	
H8B	0.8520	0.7724	0.8484	0.032*	
H8C	0.8994	0.8216	0.7804	0.032*	
C9	0.68745 (14)	0.80283 (10)	0.75108 (10)	0.0206 (4)	
H9A	0.6683	0.8044	0.8052	0.031*	
H9B	0.6240	0.7871	0.7191	0.031*	
H9C	0.7108	0.8531	0.7350	0.031*	
C10	0.74430 (15)	0.66745 (10)	0.76792 (10)	0.0211 (4)	
H10A	0.8033	0.6312	0.7613	0.032*	
H10B	0.6801	0.6515	0.7370	0.032*	
H10C	0.7266	0.6695	0.8223	0.032*	
C11	1.07286 (13)	0.77942 (9)	0.54726 (9)	0.0148 (3)	
C12	1.08302 (14)	0.86593 (10)	0.55278 (11)	0.0221 (4)	
H12A	1.0340	0.8894	0.5140	0.033*	
H12B	1.1580	0.8809	0.5437	0.033*	
H12C	1.0636	0.8826	0.6042	0.033*	
C13	1.10819 (14)	0.75406 (11)	0.46817 (10)	0.0226 (4)	
H13A	1.0609	0.7773	0.4281	0.034*	
H13B	1.1029	0.6989	0.4643	0.034*	
H13C	1.1834	0.7697	0.4614	0.034*	
C14	1.15020 (13)	0.74290 (10)	0.60857 (10)	0.0186 (4)	
H14A	1.1375	0.6883	0.6098	0.028*	
H14B	1.1367	0.7645	0.6591	0.028*	
H14C	1.2256	0.7527	0.5958	0.028*	
C15	0.71711 (12)	0.66741 (9)	0.46187 (9)	0.0131 (3)	
H15	0.7509	0.6624	0.4141	0.016*	
C16	0.56431 (13)	0.60475 (9)	0.40459 (9)	0.0122 (3)	
C17	0.60834 (13)	0.60514 (9)	0.32927 (9)	0.0140 (3)	
C18	0.46527 (12)	0.57224 (9)	0.41737 (9)	0.0119 (3)	
C19	0.40078 (13)	0.53784 (9)	0.35600 (9)	0.0141 (3)	
C20	0.33269 (12)	0.55019 (9)	0.50908 (9)	0.0130 (3)	
H20	0.2899	0.5258	0.4697	0.016*	
C21	0.28904 (12)	0.55523 (9)	0.58319 (9)	0.0122 (3)	
C22	0.18261 (13)	0.52592 (9)	0.59065 (9)	0.0144 (3)	
H22	0.1463	0.5038	0.5469	0.017*	
C23	0.13077 (13)	0.52844 (9)	0.65864 (9)	0.0139 (3)	
C24	0.18870 (13)	0.56085 (9)	0.72222 (10)	0.0153 (3)	
H24	0.1538	0.5625	0.7699	0.018*	
C25	0.29211 (13)	0.59022 (9)	0.72021 (9)	0.0143 (3)	
C26	0.34663 (13)	0.58787 (9)	0.64890 (9)	0.0137 (3)	
C27	0.01364 (13)	0.50025 (10)	0.66406 (10)	0.0170 (3)	
C28	0.00085 (18)	0.42155 (12)	0.63012 (14)	0.0377 (5)	
H28A	0.0470	0.3861	0.6599	0.056*	
H28B	0.0225	0.4220	0.5766	0.056*	
H28C	−0.0752	0.4057	0.6320	0.056*	
C29	−0.02449 (15)	0.49953 (13)	0.74654 (11)	0.0312 (5)	
H29A	0.0230	0.4666	0.7783	0.047*	
H29B	−0.0992	0.4806	0.7468	0.047*	
H29C	−0.0217	0.5509	0.7675	0.047*	
C30	−0.06285 (15)	0.55409 (12)	0.61742 (12)	0.0287 (4)	
H30A	−0.1381	0.5372	0.6214	0.043*	
H30B	−0.0437	0.5538	0.5633	0.043*	
H30C	−0.0552	0.6054	0.6380	0.043*	
C31	0.34962 (14)	0.62514 (10)	0.79195 (10)	0.0175 (3)	
C32	0.27723 (16)	0.62444 (13)	0.86125 (11)	0.0293 (4)	
H32A	0.2104	0.6527	0.8487	0.044*	
H32B	0.3159	0.6480	0.9054	0.044*	
H32C	0.2590	0.5722	0.8740	0.044*	
C33	0.45222 (14)	0.57956 (10)	0.81404 (10)	0.0213 (4)	
H33A	0.4881	0.6017	0.8601	0.032*	
H33B	0.5018	0.5808	0.7715	0.032*	
H33C	0.4322	0.5271	0.8246	0.032*	
C34	0.37892 (15)	0.70810 (10)	0.77625 (11)	0.0227 (4)	
H34A	0.3126	0.7366	0.7629	0.034*	
H34B	0.4280	0.7105	0.7335	0.034*	
H34C	0.4147	0.7300	0.8224	0.034*	
C35	0.44163 (17)	0.86449 (11)	0.59519 (11)	0.0272 (4)	
H35A	0.3795	0.8573	0.6277	0.041*	
H35B	0.4546	0.9186	0.5883	0.041*	
H35C	0.5062	0.8411	0.6198	0.041*	
C36	0.53942 (15)	0.84257 (11)	0.46161 (12)	0.0260 (4)	
H36A	0.5411	0.8186	0.4109	0.039*	
H36B	0.5994	0.8232	0.4947	0.039*	
H36C	0.5469	0.8974	0.4560	0.039*	
C37	0.71611 (16)	0.99001 (11)	0.57521 (12)	0.0284 (4)	
C38	0.82087 (17)	0.96856 (14)	0.60957 (14)	0.0398 (5)	
H38A	0.8254	0.9844	0.6636	0.060*	
H38B	0.8786	0.9932	0.5818	0.060*	
H38C	0.8294	0.9136	0.6066	0.060*	

### Atomic displacement parameters (Å^2^)

**Table:**

	*U*^11^	*U*^22^	*U*^33^	*U*^12^	*U*^13^	*U*^23^
Zn	0.01068 (9)	0.01551 (10)	0.01115 (10)	−0.00142 (7)	0.00141 (7)	−0.00244 (7)
S1	0.01486 (19)	0.0156 (2)	0.0185 (2)	−0.00009 (15)	−0.00009 (15)	−0.00080 (16)
O1	0.0123 (6)	0.0290 (7)	0.0141 (6)	−0.0051 (5)	0.0029 (4)	−0.0070 (5)
O2	0.0122 (5)	0.0260 (7)	0.0120 (6)	−0.0043 (5)	0.0008 (4)	0.0004 (5)
O3	0.0160 (6)	0.0143 (6)	0.0291 (7)	0.0003 (5)	−0.0007 (5)	−0.0002 (5)
N1	0.0125 (6)	0.0148 (7)	0.0112 (7)	0.0002 (5)	−0.0010 (5)	−0.0006 (5)
N2	0.0133 (6)	0.0126 (7)	0.0105 (7)	0.0012 (5)	0.0014 (5)	0.0003 (5)
N3	0.0248 (8)	0.0260 (8)	0.0167 (8)	−0.0025 (6)	0.0036 (6)	−0.0005 (6)
N4	0.0203 (8)	0.0251 (8)	0.0207 (8)	−0.0033 (6)	−0.0005 (6)	−0.0046 (6)
N5	0.0405 (11)	0.0296 (11)	0.0778 (16)	−0.0031 (9)	−0.0226 (11)	0.0054 (10)
C1	0.0124 (7)	0.0145 (8)	0.0130 (8)	0.0003 (6)	0.0007 (6)	−0.0011 (6)
C2	0.0135 (7)	0.0121 (8)	0.0125 (8)	0.0012 (6)	0.0002 (6)	−0.0018 (6)
C3	0.0139 (7)	0.0134 (8)	0.0132 (8)	−0.0004 (6)	−0.0015 (6)	−0.0020 (6)
C4	0.0120 (7)	0.0132 (8)	0.0131 (8)	0.0007 (6)	0.0014 (6)	0.0019 (6)
C5	0.0129 (7)	0.0150 (8)	0.0109 (8)	0.0023 (6)	0.0018 (6)	0.0013 (6)
C6	0.0126 (7)	0.0141 (8)	0.0122 (8)	0.0004 (6)	0.0002 (6)	0.0006 (6)
C7	0.0135 (8)	0.0169 (8)	0.0113 (8)	0.0004 (6)	0.0013 (6)	−0.0024 (6)
C8	0.0187 (9)	0.0323 (10)	0.0125 (8)	−0.0032 (7)	−0.0005 (7)	−0.0052 (7)
C9	0.0215 (9)	0.0236 (10)	0.0169 (9)	0.0038 (7)	0.0037 (7)	−0.0046 (7)
C10	0.0271 (9)	0.0213 (9)	0.0154 (9)	−0.0026 (7)	0.0060 (7)	0.0006 (7)
C11	0.0118 (7)	0.0191 (8)	0.0135 (8)	−0.0022 (6)	0.0020 (6)	0.0004 (7)
C12	0.0181 (8)	0.0208 (9)	0.0274 (10)	−0.0044 (7)	0.0011 (7)	0.0040 (8)
C13	0.0136 (8)	0.0375 (11)	0.0171 (9)	−0.0064 (7)	0.0040 (7)	−0.0040 (8)
C14	0.0124 (8)	0.0231 (9)	0.0203 (9)	0.0004 (7)	0.0017 (7)	0.0024 (7)
C15	0.0135 (7)	0.0147 (8)	0.0112 (8)	0.0020 (6)	0.0017 (6)	0.0010 (6)
C16	0.0144 (7)	0.0121 (8)	0.0100 (8)	0.0020 (6)	0.0001 (6)	−0.0003 (6)
C17	0.0127 (7)	0.0145 (8)	0.0146 (8)	−0.0008 (6)	−0.0014 (6)	−0.0007 (6)
C18	0.0138 (7)	0.0110 (8)	0.0108 (7)	0.0013 (6)	0.0000 (6)	0.0005 (6)
C19	0.0139 (7)	0.0149 (8)	0.0138 (8)	0.0007 (6)	0.0033 (6)	0.0002 (6)
C20	0.0144 (7)	0.0108 (8)	0.0138 (8)	0.0004 (6)	−0.0010 (6)	−0.0010 (6)
C21	0.0125 (7)	0.0118 (8)	0.0125 (8)	0.0010 (6)	0.0009 (6)	0.0011 (6)
C22	0.0150 (8)	0.0135 (8)	0.0145 (8)	−0.0008 (6)	−0.0003 (6)	0.0001 (6)
C23	0.0131 (7)	0.0126 (8)	0.0162 (8)	0.0002 (6)	0.0018 (6)	0.0029 (6)
C24	0.0167 (8)	0.0166 (8)	0.0129 (8)	0.0007 (6)	0.0038 (6)	−0.0003 (6)
C25	0.0160 (8)	0.0149 (8)	0.0118 (8)	0.0017 (6)	0.0005 (6)	0.0001 (6)
C26	0.0136 (7)	0.0134 (8)	0.0140 (8)	0.0013 (6)	−0.0006 (6)	0.0022 (6)
C27	0.0166 (8)	0.0175 (9)	0.0172 (8)	−0.0054 (6)	0.0055 (6)	−0.0020 (7)
C28	0.0358 (12)	0.0261 (11)	0.0527 (14)	−0.0109 (9)	0.0218 (10)	−0.0110 (10)
C29	0.0212 (9)	0.0500 (13)	0.0228 (10)	−0.0085 (9)	0.0055 (8)	0.0026 (9)
C30	0.0181 (9)	0.0363 (12)	0.0316 (11)	−0.0033 (8)	−0.0012 (8)	0.0057 (9)
C31	0.0181 (8)	0.0221 (9)	0.0125 (8)	−0.0027 (7)	0.0018 (6)	−0.0027 (7)
C32	0.0260 (10)	0.0454 (12)	0.0168 (9)	−0.0096 (9)	0.0045 (8)	−0.0105 (9)
C33	0.0236 (9)	0.0263 (10)	0.0136 (8)	−0.0007 (7)	−0.0037 (7)	0.0002 (7)
C34	0.0257 (9)	0.0225 (9)	0.0196 (9)	−0.0002 (7)	−0.0022 (7)	−0.0049 (7)
C35	0.0344 (11)	0.0256 (10)	0.0215 (10)	−0.0044 (8)	0.0019 (8)	−0.0061 (8)
C36	0.0213 (9)	0.0274 (10)	0.0300 (10)	−0.0052 (8)	0.0092 (8)	−0.0012 (8)
C37	0.0312 (11)	0.0192 (10)	0.0345 (11)	−0.0027 (8)	−0.0034 (9)	0.0023 (8)
C38	0.0298 (11)	0.0479 (14)	0.0412 (13)	0.0011 (10)	−0.0056 (10)	0.0119 (11)

### Geometric parameters (Å, °)

**Table:**

Zn—O1	1.9470 (11)	C14—H14C	0.9800
Zn—O2	1.9390 (11)	C15—H15	0.9500
Zn—O3	2.0467 (12)	C16—C18	1.377 (2)
Zn—N1	2.0939 (14)	C16—C17	1.439 (2)
Zn—N2	2.1001 (13)	C18—C19	1.439 (2)
S1—O3	1.5155 (12)	C20—C21	1.421 (2)
S1—C35	1.7803 (19)	C20—H20	0.9500
S1—C36	1.7826 (18)	C21—C22	1.422 (2)
O1—C1	1.2919 (19)	C21—C26	1.440 (2)
O2—C26	1.2950 (19)	C22—C23	1.369 (2)
N1—C15	1.317 (2)	C22—H22	0.9500
N1—C16	1.386 (2)	C23—C24	1.412 (2)
N2—C20	1.310 (2)	C23—C27	1.535 (2)
N2—C18	1.382 (2)	C24—C25	1.379 (2)
N3—C17	1.146 (2)	C24—H24	0.9500
N4—C19	1.147 (2)	C25—C26	1.436 (2)
N5—C37	1.131 (3)	C25—C31	1.538 (2)
C1—C6	1.439 (2)	C27—C28	1.519 (3)
C1—C2	1.446 (2)	C27—C29	1.529 (2)
C2—C3	1.380 (2)	C27—C30	1.546 (3)
C2—C7	1.535 (2)	C28—H28A	0.9800
C3—C4	1.421 (2)	C28—H28B	0.9800
C3—H3	0.9500	C28—H28C	0.9800
C4—C5	1.367 (2)	C29—H29A	0.9800
C4—C11	1.533 (2)	C29—H29B	0.9800
C5—C6	1.428 (2)	C29—H29C	0.9800
C5—H5	0.9500	C30—H30A	0.9800
C6—C15	1.421 (2)	C30—H30B	0.9800
C7—C8	1.533 (2)	C30—H30C	0.9800
C7—C9	1.536 (2)	C31—C32	1.530 (2)
C7—C10	1.539 (2)	C31—C33	1.535 (2)
C8—H8A	0.9800	C31—C34	1.540 (3)
C8—H8B	0.9800	C32—H32A	0.9800
C8—H8C	0.9800	C32—H32B	0.9800
C9—H9A	0.9800	C32—H32C	0.9800
C9—H9B	0.9800	C33—H33A	0.9800
C9—H9C	0.9800	C33—H33B	0.9800
C10—H10A	0.9800	C33—H33C	0.9800
C10—H10B	0.9800	C34—H34A	0.9800
C10—H10C	0.9800	C34—H34B	0.9800
C11—C13	1.528 (2)	C34—H34C	0.9800
C11—C12	1.540 (2)	C35—H35A	0.9800
C11—C14	1.542 (2)	C35—H35B	0.9800
C12—H12A	0.9800	C35—H35C	0.9800
C12—H12B	0.9800	C36—H36A	0.9800
C12—H12C	0.9800	C36—H36B	0.9800
C13—H13A	0.9800	C36—H36C	0.9800
C13—H13B	0.9800	C37—C38	1.450 (3)
C13—H13C	0.9800	C38—H38A	0.9800
C14—H14A	0.9800	C38—H38B	0.9800
C14—H14B	0.9800	C38—H38C	0.9800
			
O2—Zn—O1	97.38 (5)	N3—C17—C16	176.04 (18)
O2—Zn—O3	103.71 (5)	C16—C18—N2	117.53 (14)
O1—Zn—O3	109.57 (5)	C16—C18—C19	121.55 (14)
O2—Zn—N1	150.43 (5)	N2—C18—C19	120.86 (14)
O1—Zn—N1	88.26 (5)	N4—C19—C18	174.84 (17)
O3—Zn—N1	101.60 (5)	N2—C20—C21	124.94 (14)
O2—Zn—N2	87.04 (5)	N2—C20—H20	117.5
O1—Zn—N2	159.88 (5)	C21—C20—H20	117.5
O3—Zn—N2	88.21 (5)	C20—C21—C22	116.52 (14)
N1—Zn—N2	78.69 (5)	C20—C21—C26	123.51 (14)
O3—S1—C35	106.31 (8)	C22—C21—C26	119.97 (14)
O3—S1—C36	106.12 (8)	C23—C22—C21	122.24 (15)
C35—S1—C36	98.09 (9)	C23—C22—H22	118.9
C1—O1—Zn	132.84 (10)	C21—C22—H22	118.9
C26—O2—Zn	128.42 (10)	C22—C23—C24	116.77 (15)
S1—O3—Zn	138.77 (7)	C22—C23—C27	121.18 (15)
C15—N1—C16	122.47 (14)	C24—C23—C27	122.00 (14)
C15—N1—Zn	125.65 (11)	C25—C24—C23	124.81 (15)
C16—N1—Zn	111.62 (10)	C25—C24—H24	117.6
C20—N2—C18	122.86 (14)	C23—C24—H24	117.6
C20—N2—Zn	123.57 (11)	C24—C25—C26	118.56 (15)
C18—N2—Zn	111.52 (10)	C24—C25—C31	121.73 (14)
O1—C1—C6	123.11 (14)	C26—C25—C31	119.71 (14)
O1—C1—C2	119.20 (14)	O2—C26—C25	119.29 (14)
C6—C1—C2	117.68 (14)	O2—C26—C21	123.08 (14)
C3—C2—C1	118.19 (14)	C25—C26—C21	117.63 (14)
C3—C2—C7	121.84 (14)	C28—C27—C29	109.03 (16)
C1—C2—C7	119.93 (14)	C28—C27—C23	110.91 (15)
C2—C3—C4	124.96 (15)	C29—C27—C23	112.83 (14)
C2—C3—H3	117.5	C28—C27—C30	108.11 (16)
C4—C3—H3	117.5	C29—C27—C30	107.01 (15)
C5—C4—C3	116.58 (14)	C23—C27—C30	108.77 (14)
C5—C4—C11	124.38 (14)	C27—C28—H28A	109.5
C3—C4—C11	119.00 (14)	C27—C28—H28B	109.5
C4—C5—C6	122.44 (15)	H28A—C28—H28B	109.5
C4—C5—H5	118.8	C27—C28—H28C	109.5
C6—C5—H5	118.8	H28A—C28—H28C	109.5
C15—C6—C5	116.26 (14)	H28B—C28—H28C	109.5
C15—C6—C1	123.81 (14)	C27—C29—H29A	109.5
C5—C6—C1	119.87 (14)	C27—C29—H29B	109.5
C8—C7—C2	111.26 (13)	H29A—C29—H29B	109.5
C8—C7—C9	107.46 (14)	C27—C29—H29C	109.5
C2—C7—C9	110.91 (13)	H29A—C29—H29C	109.5
C8—C7—C10	107.55 (14)	H29B—C29—H29C	109.5
C2—C7—C10	110.07 (13)	C27—C30—H30A	109.5
C9—C7—C10	109.50 (14)	C27—C30—H30B	109.5
C7—C8—H8A	109.5	H30A—C30—H30B	109.5
C7—C8—H8B	109.5	C27—C30—H30C	109.5
H8A—C8—H8B	109.5	H30A—C30—H30C	109.5
C7—C8—H8C	109.5	H30B—C30—H30C	109.5
H8A—C8—H8C	109.5	C32—C31—C33	107.50 (15)
H8B—C8—H8C	109.5	C32—C31—C25	111.81 (14)
C7—C9—H9A	109.5	C33—C31—C25	109.75 (14)
C7—C9—H9B	109.5	C32—C31—C34	107.25 (15)
H9A—C9—H9B	109.5	C33—C31—C34	110.41 (14)
C7—C9—H9C	109.5	C25—C31—C34	110.06 (14)
H9A—C9—H9C	109.5	C31—C32—H32A	109.5
H9B—C9—H9C	109.5	C31—C32—H32B	109.5
C7—C10—H10A	109.5	H32A—C32—H32B	109.5
C7—C10—H10B	109.5	C31—C32—H32C	109.5
H10A—C10—H10B	109.5	H32A—C32—H32C	109.5
C7—C10—H10C	109.5	H32B—C32—H32C	109.5
H10A—C10—H10C	109.5	C31—C33—H33A	109.5
H10B—C10—H10C	109.5	C31—C33—H33B	109.5
C13—C11—C4	111.82 (13)	H33A—C33—H33B	109.5
C13—C11—C12	108.83 (14)	C31—C33—H33C	109.5
C4—C11—C12	109.44 (13)	H33A—C33—H33C	109.5
C13—C11—C14	107.92 (14)	H33B—C33—H33C	109.5
C4—C11—C14	109.64 (13)	C31—C34—H34A	109.5
C12—C11—C14	109.13 (14)	C31—C34—H34B	109.5
C11—C12—H12A	109.5	H34A—C34—H34B	109.5
C11—C12—H12B	109.5	C31—C34—H34C	109.5
H12A—C12—H12B	109.5	H34A—C34—H34C	109.5
C11—C12—H12C	109.5	H34B—C34—H34C	109.5
H12A—C12—H12C	109.5	S1—C35—H35A	109.5
H12B—C12—H12C	109.5	S1—C35—H35B	109.5
C11—C13—H13A	109.5	H35A—C35—H35B	109.5
C11—C13—H13B	109.5	S1—C35—H35C	109.5
H13A—C13—H13B	109.5	H35A—C35—H35C	109.5
C11—C13—H13C	109.5	H35B—C35—H35C	109.5
H13A—C13—H13C	109.5	S1—C36—H36A	109.5
H13B—C13—H13C	109.5	S1—C36—H36B	109.5
C11—C14—H14A	109.5	H36A—C36—H36B	109.5
C11—C14—H14B	109.5	S1—C36—H36C	109.5
H14A—C14—H14B	109.5	H36A—C36—H36C	109.5
C11—C14—H14C	109.5	H36B—C36—H36C	109.5
H14A—C14—H14C	109.5	N5—C37—C38	179.9 (3)
H14B—C14—H14C	109.5	C37—C38—H38A	109.5
N1—C15—C6	125.57 (15)	C37—C38—H38B	109.5
N1—C15—H15	117.2	H38A—C38—H38B	109.5
C6—C15—H15	117.2	C37—C38—H38C	109.5
C18—C16—N1	117.67 (14)	H38A—C38—H38C	109.5
C18—C16—C17	121.36 (14)	H38B—C38—H38C	109.5
N1—C16—C17	120.90 (14)		
			
O2—Zn—O1—C1	150.82 (15)	C3—C4—C11—C12	−69.76 (19)
O3—Zn—O1—C1	−101.74 (15)	C5—C4—C11—C14	−127.48 (17)
N1—Zn—O1—C1	−0.04 (15)	C3—C4—C11—C14	49.91 (19)
N2—Zn—O1—C1	49.2 (2)	C16—N1—C15—C6	−178.57 (15)
O1—Zn—O2—C26	166.41 (14)	Zn—N1—C15—C6	7.9 (2)
O3—Zn—O2—C26	54.13 (14)	C5—C6—C15—N1	177.20 (15)
N1—Zn—O2—C26	−94.02 (16)	C1—C6—C15—N1	0.0 (3)
N2—Zn—O2—C26	−33.31 (14)	C15—N1—C16—C18	173.74 (15)
C35—S1—O3—Zn	−60.18 (14)	Zn—N1—C16—C18	−11.89 (17)
C36—S1—O3—Zn	43.54 (14)	C15—N1—C16—C17	−9.4 (2)
O2—Zn—O3—S1	119.64 (12)	Zn—N1—C16—C17	164.96 (12)
O1—Zn—O3—S1	16.51 (13)	C18—C16—C17—N3	142 (2)
N1—Zn—O3—S1	−75.78 (12)	N1—C16—C17—N3	−34 (3)
N2—Zn—O3—S1	−153.85 (12)	N1—C16—C18—N2	−1.0 (2)
O2—Zn—N1—C15	−108.97 (15)	C17—C16—C18—N2	−177.83 (14)
O1—Zn—N1—C15	−7.03 (13)	N1—C16—C18—C19	176.23 (14)
O3—Zn—N1—C15	102.59 (13)	C17—C16—C18—C19	−0.6 (2)
N2—Zn—N1—C15	−171.63 (14)	C20—N2—C18—C16	177.49 (14)
O2—Zn—N1—C16	76.88 (14)	Zn—N2—C18—C16	13.30 (17)
O1—Zn—N1—C16	178.82 (11)	C20—N2—C18—C19	0.2 (2)
O3—Zn—N1—C16	−71.56 (11)	Zn—N2—C18—C19	−163.95 (12)
N2—Zn—N1—C16	14.22 (10)	C16—C18—C19—N4	−144 (2)
O2—Zn—N2—C20	27.25 (13)	N2—C18—C19—N4	33 (2)
O1—Zn—N2—C20	130.72 (15)	C18—N2—C20—C21	−178.19 (15)
O3—Zn—N2—C20	−76.57 (13)	Zn—N2—C20—C21	−15.9 (2)
N1—Zn—N2—C20	−178.78 (13)	N2—C20—C21—C22	177.32 (15)
O2—Zn—N2—C18	−168.69 (11)	N2—C20—C21—C26	−2.7 (3)
O1—Zn—N2—C18	−65.23 (19)	C20—C21—C22—C23	−178.94 (15)
O3—Zn—N2—C18	87.48 (11)	C26—C21—C22—C23	1.0 (2)
N1—Zn—N2—C18	−14.73 (10)	C21—C22—C23—C24	−0.9 (2)
Zn—O1—C1—C6	6.6 (2)	C21—C22—C23—C27	176.69 (15)
Zn—O1—C1—C2	−173.09 (11)	C22—C23—C24—C25	0.5 (2)
O1—C1—C2—C3	−174.06 (15)	C27—C23—C24—C25	−176.99 (16)
C6—C1—C2—C3	6.2 (2)	C23—C24—C25—C26	−0.4 (3)
O1—C1—C2—C7	8.2 (2)	C23—C24—C25—C31	179.57 (15)
C6—C1—C2—C7	−171.50 (14)	Zn—O2—C26—C25	−153.79 (12)
C1—C2—C3—C4	−3.7 (2)	Zn—O2—C26—C21	26.7 (2)
C7—C2—C3—C4	173.99 (15)	C24—C25—C26—O2	−179.06 (15)
C2—C3—C4—C5	−0.4 (2)	C31—C25—C26—O2	1.0 (2)
C2—C3—C4—C11	−177.95 (15)	C24—C25—C26—C21	0.5 (2)
C3—C4—C5—C6	1.7 (2)	C31—C25—C26—C21	−179.45 (14)
C11—C4—C5—C6	179.12 (15)	C20—C21—C26—O2	−1.3 (2)
C4—C5—C6—C15	−176.22 (15)	C22—C21—C26—O2	178.71 (15)
C4—C5—C6—C1	1.1 (2)	C20—C21—C26—C25	179.17 (15)
O1—C1—C6—C15	−7.7 (3)	C22—C21—C26—C25	−0.8 (2)
C2—C1—C6—C15	172.05 (15)	C22—C23—C27—C28	50.7 (2)
O1—C1—C6—C5	175.20 (15)	C24—C23—C27—C28	−131.85 (18)
C2—C1—C6—C5	−5.1 (2)	C22—C23—C27—C29	173.39 (16)
C3—C2—C7—C8	−2.6 (2)	C24—C23—C27—C29	−9.2 (2)
C1—C2—C7—C8	175.02 (15)	C22—C23—C27—C30	−68.0 (2)
C3—C2—C7—C9	116.96 (17)	C24—C23—C27—C30	109.38 (18)
C1—C2—C7—C9	−65.43 (19)	C24—C25—C31—C32	−1.6 (2)
C3—C2—C7—C10	−121.71 (17)	C26—C25—C31—C32	178.31 (16)
C1—C2—C7—C10	55.90 (19)	C24—C25—C31—C33	117.58 (17)
C5—C4—C11—C13	−7.8 (2)	C26—C25—C31—C33	−62.5 (2)
C3—C4—C11—C13	169.57 (15)	C24—C25—C31—C34	−120.71 (17)
C5—C4—C11—C12	112.85 (18)	C26—C25—C31—C34	59.2 (2)

## References

[bb1] Addison, A. W., Rao, T. N., Reedijk, J., van Rijn, J. & Verschoor, G. C. (1984). *J. Chem. Soc. Dalton Trans.* pp. 1349–1356.

[bb2] Brandenburg, K. (2006). *DIAMOND* Crystal Impact GbR, Bonn, Germany.

[bb3] Bruker (2008). *APEX2* and *SAINT* Bruker AXS Inc., Madison, Wisconsin, USA.

[bb4] Chen, L., Qiao, J., Xie, J., Duan, L., Zhang, D., Wang, L. & Qiu, Y. (2009). *Inorg. Chim. Acta*, **362**, 2327–2333.

[bb5] Farrugia, L. J. (1997). *J. Appl. Cryst.* **30**, 565.

[bb6] Liuzzo, V., Oberhauser, W. & Pucci, A. (2010). *Inorg. Chem. Commun.* **13**, 686–688.

[bb7] MacLachlan, M. J., Park, M. K. & Thompson, L. K. (1996). *Inorg. Chem.* **35**, 5492–5499. 10.1021/ic960237p 11666735

[bb8] Sheldrick, G. M. (1996). *SADABS* University of Göttingen, Germany.

[bb9] Sheldrick, G. M. (2008). *Acta Cryst.* A**64**, 112–122. 10.1107/S0108767307043930 18156677

[bb10] Shirai, K., Matsuoka, M. & Fukunishi, K. (2000). *Dyes Pigments*, **47**, 107–115.

[bb11] Spek, A. L. (2009). *Acta Cryst.* D**65**, 148–155. 10.1107/S090744490804362X PMC2631630 19171970

[bb12] Westrip, S. P. (2010). *J. Appl. Cryst.* **43**, 920–925.

